# Acid base and blood gas analysis in term neonates immediately after birth with uncomplicated neonatal transition

**DOI:** 10.1186/s12887-022-03324-z

**Published:** 2022-05-12

**Authors:** Nariae Baik-Schneditz, Bernhard Schwaberger, Berndt Urlesberger, Christina Helene Wolfsberger, Marlies Bruckner, Gerhard Pichler

**Affiliations:** 1 Division of Neonatology, Department of Paediatrics and Adolescent Medicine, Medical University of Graz, Auenbruggerplatz 34/2, 8036, Graz, Styria Austria; 2 Research Unit for Neonatal Micro- and Macrocirculation, Division of Neonatology, Medical University of Graz, Graz, Austria; 3 Research Unit for Cerebral Development and Oximetry, Division of Neonatology, Medical University of Graz, Graz, Austria

**Keywords:** Acid base, Blood gas, Term neonate, Neonatal transition, Immediately after birth

## Abstract

**Background:**

Acid base and blood gas measurements provide essential information, especially in critically ill neonates. After birth, rapidly changing physiology and difficulty to obtain blood samples represent unique challenges.

**Objectives:**

The aim of the present study was to establish normal values of capillary acid base and blood gas analysis immediately after birth in term neonates after uncomplicated neonatal transition.

**Method:**

This is a post-hoc-analysis of ancillary outcome parameter of a prospective observational study in term neonates immediately after caesarean section. Neonates were included after immediate neonatal transition without need of medical support and a capillary blood sample was taken by a heel-stick within 15–20 minutes after birth.

**Result:**

One hundred thirty-two term neonates were included with mean (SD) gestational age of 38.7 ± 0.7 weeks. The blood was drawn mean (SD) 16 ± 1.7 minutes after birth. The mean (SD) values of the analyses were: pH 7.30 ± 0.04, pCO_2_ 52.6 ± 6.4, base excess − 0.9 ± 1.7 and bicarbonate 24.8 ± 1.6.

**Conclusion:**

This is the first study describing acid base and blood gas analyses in term neonates immediately after birth with uncomplicated neonatal transition.

## Introduction

In 1957, a study group from New York, USA noticed that umbilical cord blood gas analysis may indicate preceding fetal hypoxic stress [1]. Since then, it is widely known that umbilical cord blood gas analysis might also provide important information concerning the neonate’s condition after birth [2]. Meanwhile, umbilical cord blood gas analysis is recommended in all high-risk deliveries [3, 4] and many obstetrics centers routinely perform cord blood gas analysis in all deliveries. In the last decades, there has been growing interest in research of umbilical blood gas analysis in neonates in different conditions [5, 6] – e.g. preeclamptic versus healthy preterm neonates or effect of delayed cord clamping versus early cord clamping. For precise interpretation, many study groups published normative data for umbilical cord blood gas values [7, 8].

Umbilical cord blood gas analysis provides valuable information about the neonate’s condition immediately after birth. However, the neonates’ condition may change quickly after birth, depending on the pulmonary and cardio-circulatory adaptation. Besides pulse oximetry and/or electrocardiogram monitoring, the acid base and blood gas analysis of capillary blood enables a quick point-of care information. Immediately after birth the rapidly changing physiology, the difficulty of access to arterial blood samples and the small blood volumes present unique challenges [9]. Acid base and blood gas analysis as additional information might be helpful to assess the neonate’s condition, especially if they are in need of medical support. However, to guide interventions based on blood gas analysis immediately after birth, normal values are essential.

The aim of this study was to establish normal values of acid base and blood gas values during immediate transition in term neonates without need of any medical support.

## Materials and methods

This was a post-hoc-analysis of ancillary outcome parameter of a prospective observational study conducted at the Division of Neonatology, Department of Paediatrics and Adolescent Medicine, Medical University of Graz, from October 2015 to September 2018. The prospective observational study [10] was approved by the Regional Committee on Biomedical Research Ethics (EC Number: 27–465 ex 14/15.), the decision included a possible post-hoc analysis. Informed parental consent was obtained antenatally, before neonates were included in the study.

Data of term neonates ≥37^+ 0^ gestational age born by caesarean section, who were included in the prospective observational study were eligible for this analysis. After the neonates were fully delivered, a stopwatch was started. After cord clamping, routinely performed after 30 seconds, neonates were brought to the resuscitation table and placed under an overhead heater in supine position. Routine monitoring of heart rate (HR) and arterial oxygen saturation (SpO_2_) was performed by pulse-oximetry. The data of neonates without any medical support and capillary blood sampling during immediate transition were analyzed. The capillary blood sample was taken from the heel within 15–20 minutes after birth according to the study protocol before the neonates were brought to the parents. Stabilization was performed according to the latest neonatal life support guideline recommendations [11]. The capillary blood samples were analyzed with a blood gas analyzer (ABL 800 Flex; Fa.Drott, Wiener Neustadt, Austria). The measurements of blood samples are performed on the neonatal unit of the Medical University of Graz, where only one type of blood gas analyzer has been used, whereby this analyzer is controlled regularly. The following acid base and blood gas values were recorded and analyzed: pH, partial pressure of carbon dioxide (pCO_2_), base excess and bicarbonate. As additional information hematocrit and lactate were recorded and analyzed. HR and SpO_2_, and rectal body temperature at 15 minutes after birth were assessed.

Demographic and clinical characteristics are presented as mean ± standard deviation (SD) for normally distributed continuous variables and medians with interquartile range (IQR) when the distribution was skewed. PH, pCO_2_, base excess, bicarbonate, hematocrit and lactate are presented as mean ± standard deviation (SD). The statistical analyses were performed using IBM SPSS Statistics 26.0.0 (IBM Corporation, Armonk, NY, USA).

## Results

Data of 132 term neonates were analyzed. Demographic and clinical characteristics of the study population are presented in Table 1.

**Table 1:** Demographic and clinical characteristics of the study population at 15 minute after birth

	Study population (*n* = 132)
Gestational age (weeks) – mean (SD)	38.7 ± 0.7
Birth weight (g) – mean (SD)	3260 ± 472
Female sex – n (%)	57 (43.2)
Apgar score at 5 minutes –median (IQR)	10 (10–10)
Apgar score at 10 minutes –median (IQR)	10 (10–10)
Umbilical artery pH –mean (SD)	7.31 ± 0.04
Umbilical artery CO_2_(mmHg) – mean (SD)	54.2 ± 6.8
Umbilical artery lactate (mmol/L) – mean (SD)	1.8 ± 0.7
Body temperature (°C) – mean (SD)	37.2 ± 0.34
Heart rate (beat per minute) – mean (SD)	156 ± 18
SpO_2_ (%) – mean (SD)	96 ± 2.9

The blood samples were drawn mean (SD) 16 ± 1.8 minutes after birth.

The blood samples were drawn mean (SD) 16 ± 1.8 minutes after birth. Table 2 presents the data of the mean with standard deviation (SD) and median with confidence interval (CI) of pH, pCO_2_, base excess bicarbonate, hematocrit and lactate of the capillary blood gas analyses in term infants after uncomplicated immediate neonatal transition.

**Table 2:** Capillary acid base and blood gas values within 15–20 minutes after birth in stable neonates without any medical support

	mean	SD	median	CI
pH	7.30	0.04	7.30	7.26–7.33
pCO_2_ (mmHg)	52.6	6,4	52.5	47.6–56.7
base excess (mmol/L)	−0,9	1,7	−0.9	−1.8 - 0.4
bicarbonate (mmol/L)	24,8	1,6	24.9	23.7–25.9
hematocrit (%)	57,4	8,1	57.1	53.7–61.3
lactate (mmol/l)	2,7	0,8	2.6	2.1–3.1

## Discussion/conclusion

To the best of our knowledge, this is the first description of capillary acid base and blood gas values immediately after birth in stable neonates without any medical support.

Postnatal immediate transition is a vulnerable time period, where the neonates undergo complex changes affecting all vital organ systems. If disturbances occur during this period, this might lead to severe consequences. Non-invasive monitoring of HR and SpO_2_ by pulse oximetry or electrocardiogram is recommended in neonates during stabilization immediately after birth. For this routine non-invasive monitoring, normative data are already established [12, 13]. But non-invasive monitoring often does not provide the whole information needed to judge on the cardio-respiratory status of a neonate. Acid base and blood gas analyses from capillary blood samples might give further information to guide the respiratory and medical support, especially in critically ill neonates [14]. Cousineaua et al. published reference values of capillary blood gases in term neonates at the age of 48 hours after birth. The mean pH was 7.39 and pCO_2_ was 38.7 mmHg. These values are comparable with reference values, which are considered as normal at clinical aspects [14].

Within the present study, we observed lower pH and higher pCO_2_ values compared to the published reference values in term neonates at the age of 48 hours after birth. As our study population did not need any respiratory support and returned to their parents without any sign of respiratory distress, these observed values might be physiological during fetal to neonatal transition immediately after birth.

Several studies compared blood gas values from arterial and capillary blood samples [9, 15]. Saili et al. observed in 51 neonates with moderate asphyxia 60 hours after birth, higher capillary pCO_2_ compared to arterial pCO_2_. This study group concluded, that the capillary pCO_2_ is of little use to predict arterial pCO_2_ [15]. According to the literature caution should be used, when clinicians make decision based only on capillary blood gas analyses [9, 15]. However, in neonates and especially during immediate transition, arterial blood samples are often not available and capillary blood samples are the only way to get information of the blood gases to guide respiratory or cardio-circulatory support.

We recognize some limitations in our study. First, we analyzed only capillary blood samples. Second, all included neonates were term neonates, who were observed by a neonatologist for 10–15 minutes after cesarean section. Values after spontaneous vaginal delivery might differ. Third, umbilical cord was clamped routinely after 30 seconds and values might differ in neonates with delayed umbilical cord clamping or physiological based cord clamping.

This is descriptive data of capillary acid base and blood gas values immediately after the birth of healthy term neonates after uncomplicated neonatal transition after caesarian section. The presented acid base and blood gas values can be considered as normative values for a capillary blood sample about 15–20 minutes after birth. Especially, if for some reason cord blood values are not available, this data might be of great value. Major deviations from these values might be interpreted as potentially pathological and should lead to a re-evaluation of the newborn.

## Data Availability

The datasets used and analysed during the current study are available from the corresponding author on reasonable request.

## References

[1] James LS, Weisbrot IM, Prince CE, Holaday DA, Apgar V (1958). The acid-base status of human infants in relation to birth asphyxia and the onset of respiration. J Pediatr.

[2] Armstrong L, Stenson BJ (2007). Use of umbilical cord blood gas analysis in the assessment of the newborn. Arch Dis Child Fetal Neonatal Ed.

[3] ACOG Committee on Obstetric Practice (2006). ACOG Committee Opinion No. 348, November 2006: Umbilical cord blood gas and acid-base analysis. Obstet Gynecol.

[4] Ferreira CS, Melo Â, Fachada AH, Solheiro H, Nogueira Martins N (2018). Umbilical Cord Blood Gas Analysis, Obstetric Performance and Perinatal Outcome. Rev Bras Ginecol Obstet.

[5] Sheikh M, Zoham MH, Hantoushzadeh S, Shariat M, Dalili H, Amini E (2016). Umbilical blood gas analysis in preeclamptic versus healthy pregnancies with preterm birth. J Matern Fetal Neonatal Med.

[6] De Paco C, Florido J, Garrido MC, Prados S, Navarrete L (2011). Umbilical cord blood acid-base and gas analysis after early versus delayed cord clamping in neonates at term. Arch Gynecol Obstet.

[7] Victory R, Penava D, Da Silva O, Natale R, Richardson B (2004). Umbilical cord pH and base excess values in relation to adverse outcome events for infants delivering at term. Am J Obstet Gynecol.

[8] Thorp JA, Rushing RS (1999). Umbilical cord blood gas analysis. Obstet Gynecol Clin N Am.

[9] Courtney SE, Weber KR, Breakie LA, Malin SW, Bender CV, Guo SM (1990). Capillary blood gases in the neonate. A reassessment and review of the literature. Am J Dis Child.

[10] Bresesti I, Bruckner M, Mattersberger C, Baik-Schneditz N, Schwaberger B, Mileder L (2020). Feasibilty of Transcutaneous pCO(2) Monitoring During Immediate Transition After Birth-A Prospective Observational Study. Front Pediatr.

[11] Wyllie J, Bruinenberg J, Roehr CC, Rudiger M, Trevisanuto D, Urlesberger B (2015). European Resuscitation Council Guidelines for Resuscitation 2015: Section 7. Resuscitation and support of transition of babies at birth. Resuscitation.

[12] Dawson JA, Kamlin COF, Wong C, Te Pas AB, Vento M, Cole TJ (2010). Changes in heart rate in the first minutes after birth. Archives of Disease in Childhood. Fetal Neonat Ed.

[13] Pichler G, Cheung PY, Binder C, O'Reilly M, Schwaberger B, Aziz K (2014). Time course study of blood pressure in term and preterm infants immediately after birth. PLoS One.

[14] Cousineau J, Anctil S, Carceller A, Gonthier M, Delvin EE (2005). Neonate capillary blood gas reference values. Clin Biochem.

[15] Saili A, Dutta AK, Sarna MS (1992). Reliability of capillary blood gas estimation in neonates. Indian Pediatr.

